# Soil TPH Concentration Estimation Using Vegetation Indices in an Oil Polluted Area of Eastern China

**DOI:** 10.1371/journal.pone.0054028

**Published:** 2013-01-16

**Authors:** Linhai Zhu, Xuechun Zhao, Liming Lai, Jianjian Wang, Lianhe Jiang, Jinzhi Ding, Nanxi Liu, Yunjiang Yu, Junsheng Li, Nengwen Xiao, Yuanrun Zheng, Glyn M. Rimmington

**Affiliations:** 1 Key Laboratory of Resource Plants, Beijing Botanical Garden, West China Subalpine Botanical Garden, Institute of Botany, Chinese Academy of Sciences, Xiangshan, Beijing, China; 2 University of Chinese Academy of Sciences, Beijing, China; 3 Chinese Research Academy of Environmental Sciences, Beijing, China; 4 Global Learning College of Engineering, Wichita State University, Wichita, Kansas, United States of America; DOE Pacific Northwest National Laboratory, United States of America

## Abstract

Assessing oil pollution using traditional field-based methods over large areas is difficult and expensive. Remote sensing technologies with good spatial and temporal coverage might provide an alternative for monitoring oil pollution by recording the spectral signals of plants growing in polluted soils. Total petroleum hydrocarbon concentrations of soils and the hyperspectral canopy reflectance were measured in wetlands dominated by reeds (*Phragmites australis*) around oil wells that have been producing oil for approximately 10 years in the Yellow River Delta, eastern China to evaluate the potential of vegetation indices and red edge parameters to estimate soil oil pollution. The detrimental effect of oil pollution on reed communities was confirmed by the evidence that the aboveground biomass decreased from 1076.5 g m^−2^ to 5.3 g m^−2^ with increasing total petroleum hydrocarbon concentrations ranging from 9.45 mg kg^−1^ to 652 mg kg^−1^. The modified chlorophyll absorption ratio index (MCARI) best estimated soil TPH concentration among 20 vegetation indices. The linear model involving MCARI had the highest coefficient of determination (*R* ^2^ = 0.73) and accuracy of prediction (*RMSE* = 104.2 mg kg^−1^). For other vegetation indices and red edge parameters, the *R^2^* and *RMSE* values ranged from 0.64 to 0.71 and from 120.2 mg kg^−1^ to 106.8 mg kg^−1^ respectively. The traditional broadband normalized difference vegetation index (NDVI), one of the broadband multispectral vegetation indices (BMVIs), produced a prediction (*R* ^2^ = 0.70 and *RMSE* = 110.1 mg kg^−1^) similar to that of MCARI. These results corroborated the potential of remote sensing for assessing soil oil pollution in large areas. Traditional BMVIs are still of great value in monitoring soil oil pollution when hyperspectral data are unavailable.

## Introduction

Oil is a crucial energy resource and vital industrial raw material. Most oil is produced on land [1]. With increasing industrial production, oil pollution has become a serious worldwide environmental problem, especially at the oil mining stage in the field. It has many detrimental effects on the composition, structure and functioning of terrestrial ecosystems [2], [3], [4], [5], including loss of biodiversity [6]. Oil pollutants also transfer via food chains and eventually harm human health [7]. In addition, residual oil hydrocarbons can persist in the soil for decades [3], [8] and have a chronic effect on ecosystems and human beings. Therefore, it is crucial to monitor oil pollution accurately and quickly in areas where oil is produced.

Oil pollution at the mining stage is usually monitored by field sampling of soil, water, atmosphere and vegetation, and laboratory analysis of oil pollutants. However, these methods, especially when applied in large areas, are difficult, time-consuming and expensive. Remote sensing can cover large areas simultaneously and periodically in a non-destructive fashion, and offers a valid alternative to traditional ground-based methods [9], [10], [11]. Among remote sensing data, hyperspectral data consisting of hundreds of contiguous spectral bands narrower than 10 nm throughout the visible-infrared spectrum could prove to be invaluable in developing environmental monitoring capabilities [12].

The reflectance of a leaf is governed by its pigment concentrations, morphological and anatomical properties, water content and other biochemical properties [13], [14]. Canopy reflectance of plants is determined by the optical properties of leaves, and is modified by leaf area index (LAI), the amount of green biomass, canopy architecture and leaf angle distribution [15], [16]. Alterations in these variables caused by oil pollution and other environmental stresses induce changes in canopy reflectance that can be detected by remote sensors and might be used as indicators for oil pollution of soils [10], [17], [18]. However, the spectral signal captured by sensors includes not only spectral characteristics of plants, but also optical properties of soils and air within the field of view [19], [20]. Remote sensing by sensors is also influenced by viewing angle and illumination geometry [16]. Therefore, vegetation indices (VIs) [19], [21] are proposed to enhance the spectral contribution from green plants and minimize those from soil, air and other external factors.

Vegetation indices combine two or more spectral bands to quantitatively characterize the physiological status of plants [22]. According to spectral resolution of data, vegetation indices can be classified into two classes: multispectral vegetation indices (MVIs) and hyperspectral vegetation indices (HVIs) [21]. Multispectral vegetation indices are first formulated with multispectral bands (such as those of Landsat ETM+ and NOAA AVHRR), but they can also be calculated from hyperspectral data using the reflectance at specific wavelengths [17], [21]. Multispectral vegetation indices calculated in these two fashions can be defined as broadband multispectral vegetation indices (BMVIs) and narrowband multispectral vegetation indices (NMVIs). The normalized difference vegetation index (NDVI) is one of the oldest and most widely used multispectral vegetation indices [23]. Hyperspectral vegetation indices are proposed based on the absorption and reflectance properties in specific regions of the high resolution spectrum [24], [25].

Hyperspectral remote sensing not only provides new data for the development of vegetation indices, but also can detect some characteristics that traditional multispectral remote sensing cannot. Red edge is a unique and most notable feature of green plants, and cannot be measured using spectral bands broader than 100 nm. It is a sharp change in reflectance between low red reflectance caused by chlorophyll absorption near 680 nm and high infrared reflectance governed by internal leaf scattering near 750 nm [26]. Three parameters, red edge position (REP), slope (RES) and area (REA), can be calculated using the first derivative of reflectance. Red edge position is the maximum of the first derivative of reflectance between 680 nm and 750 nm. Red edge slope and red edge area are the maximum and integral of the first derivative in this region. In addition, the linear four-point interpolation technique [27], the Gaussian function fitting technique [28] and the polynomial fitting technique [21], [29] have been proposed to parameterize the red edge.

Remote sensing has previously been used to detect stresses in plants [30], [31], [32]. Visible reflectance increased consistently in stressed leaves for eight stress agents and among six vascular plant species [30], and ratios of leaf reflectance were further evaluated as indicators of these stresses for these plants [31]. Remote sensing has been used to estimate metal pollutions in soils [17], [33]. Rosso et al reported that lightweight petroleum induced significant changes in reflectance between 500 nm and 1550 nm [18]. Current and older foliage from spruce/fir forest affected by air pollution showed an approximately 5 nm shift away from the normal value of red edge position toward shorter wavelengths [34]. A hyperspectral derivative ratio in the red edge region has been used to identify plant stress due to gas leaks before visible symptoms were observed [32]. Although vegetation indices and red edge have been employed to estimate soil metal contamination [10], [17], [33], there are very few studies concerning the estimation of soil oil pollution by remote sensing. Therefore, it is necessary to evaluate current remote sensing methods and to develop a suitable method for oil pollution monitoring.

As the youngest land and one of the largest wetlands in China, the Yellow River Delta provides many ecological functions, such as habitats for numerous species [35]. However, this region is widely subjected to serious crude oil contamination due to oil mining. Shengli oilfield, as the second largest in China, lies mainly in this region. Since oil production began in 1964, intensive exploitation of oil for decades has produced detrimental effects on wetland ecosystems. Shengli oilfield discharges more than 100 000 tons of oily sludge into soils each year [36], and oil pollution has become the main anthropogenic source of ecological risks [37]. Therefore, estimating oil pollution accurately and quickly is urgently needed to enable control of its adverse effects and protect the remaining wetlands.

In this study, the feasibility of remote sensing to estimate soil oil pollution in the Yellow River Delta, Shandong Province, eastern China was investigated, and an effective remote sensing method was developed to monitor oil pollution accurately and quickly. This will be useful for establishing an early warning system and mitigating the harmful effects of oil pollution.

## Materials and Methods

### Ethics Statements

The field study was conducted with the permission of the Hekou Oil Production Plant, Shengli Oilfield Company, SINOPEC and the owners of the lands around the oil wells. No protected plant species were present in quadrats of the plant communities.

### Site and Sample Design

**Figure 1:**
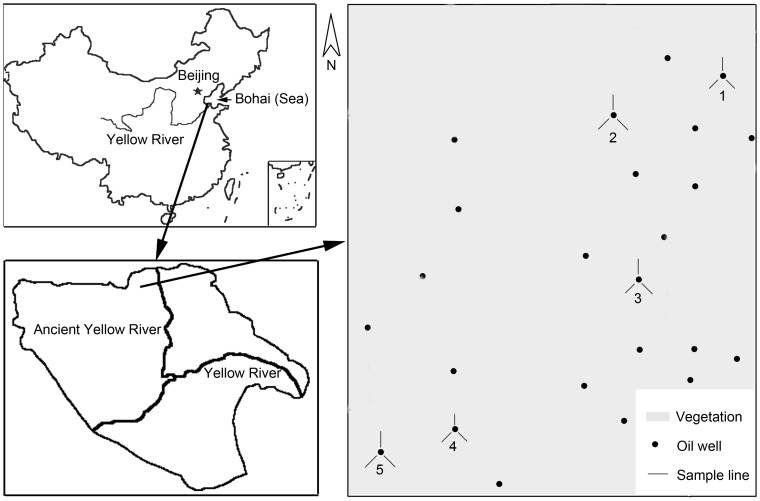
The location of the study site in eastern China. The spatial extent of the third panel is approximately 7 km by 8 km.

**Figure 2:**
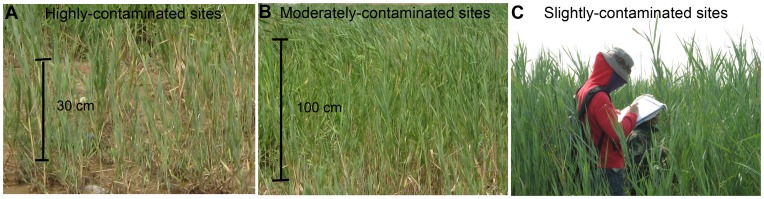
Reed communities with different oil pollution.

This study was carried out in the Chengdong Oilfield belonging to the Shengli Oilfield Company (118°34′38.35″ E-118°38′08.53″ E, 37°57′34.07″ N-38°01′28.50″ N) (Figure 1). The sites are adjacent to the ancient Yellow River, the National Natural Reserve of the Yellow River Delta and the National Forest Park of the Yellow River Estuary. The region is sparsely populated and oil exploitation is the major production activity. Reeds dominate this region and several plant species grow under the reeds (Figure 2). The soil is a saline soil.

A field study was conducted in August 2009. Sample plots around each of five oil wells and three sample plots (bare soil) away from oil wells were established. Another bare soil plot was established for testing spectroradiometer. Soil color was uniform in all study sites. The mining history of the oil wells ranged from 7 to 13 years, and had an average of 10 years, with a standard deviation of 2.5 years. All wells produced heavy oils. Three sample lines were laid out in directions away from the wells. One line was set up along the prevailing wind direction, while the other two were established at a 120° angle from the first line to represent different oil pollution conditions in different directions. Along each sample line, three 1m×1m quadrats were established around each point at different distances from each oil well, 0 m, 5m, 10m, 20m, 30m, 50m, and 100m. This sampling procedure represents possible differences in oil pollution with different distances from the oil wells. At same distance points to oil well center in three lines of each oil well, e.g. at 5 m, data of three quadrats were averaged. Therefore, there are totally seven averaged data for one well.

### Reflectance Measurement

The reflectance spectra of each quadrat and the bare soils were recorded using a field spectroradiometer ASD FieldSpec FR (Analytical Spectral Devices, Inc., Boulder, Colorado, USA). This spectroradiometer was fitted with a fiber optic probe having a 25° field of view. It covers the spectrum between 350 nm and 2500 nm and its spectral sampling interval is 1.4 nm for the region 350–1000 nm and 2 nm for the region 1000–2500 nm. Its spectral resolution (full-width-half-maximum) is 3 nm for the region 350–1000 nm and 10 nm for the region 1000–2500 nm. The spectra were then interpolated by the ASD software to produce readings at every 1 nm. Measurements were taken between 10:00 and 12:00 am on clear days without clouds and winds. Scans were taken from a height of 50 cm above the canopies of the plant communities looking towards the nadir position so that the field of view was a circular area having a diameter of about 22 cm. Ten replicates were measured for each quadrat. Before measurement of each quadrat, the radiance of a white standard panel coated with BaSO_4_ and the dark current (Systematic noise from the instrument electronics and detectors) were recorded for reference and optimization of measurement [18], [32]. Because the energy incident on plant or soil is approximately equivalent to that reflected by the white panel, the reflectance (*ρ*, %) can be calculated as follows [38].
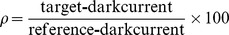
where, ‘target’ is the energy reflected off plant canopies or bare soils, and ‘reference’ is that reflected off BaSO_4_ white panel.

After the individual spectra of reflectance were inspected for bad data and outliers, they were averaged by quadrat [38]. The averaged reflectance values were used to calculate vegetation indices. The reflectances of broadbands similar to Landsat ETM+ were calculated as the average reflectance of all wavelengths in the corresponding bands of spectroradiometer ASD FieldSpec FR.

### Aboveground Biomass and Species of the Plant Communities

After measurement of canopy reflectance, the aboveground portion of plants in each quadrat was harvested. After the fresh aboveground material was weighed, it was dried at 80°C for 72 h and then reweighed. Species in every quadrat were recorded (Table 1).

**Table 1:** Species in every quadrat in study site.

Oil wells	Quadrats	Species
1	1	*Phragmites australis*
	2	*P. australis*, *Tamarix austromongolica* (Rare)
	3	*P. australis*, *Suaeda*. *salsa*
	4	*P. australis*, *S*. *salsa*, *Cynanchum chinense*
	5	*P. australis*, *S*. *salsa*, *C*. *chinense*
	6	*P. australis*, *S*. *salsa*, *Aeluropus sinensis*
	7	*P. australis*, *S*. *salsa*, *T. austromongolica* (Rare), *C*. *chinense* (Very Rare), *Setaria viridis* (Very Rare)
2	1	*P. australis*, *A. sinensis* (Rare)
	2	*P. australis*, *Scorzonera mongolica* (Rare)
	3	*P. australis*, *S*. *salsa*
	4	*P. australis*, *S*. *salsa*, *Limonium bicolor* (Very Rare)
	5	*P. australis*, *S*. *salsa*, *C*. *chinense* (Rare)
	6	*P. australis*, *S*. *salsa*, *Apocynum venetum* (Rare)
	7	*P. australis*, *S*. *salsa*, *C*. *chinense* (Rare), *S*. *viridis* (Very Rare)
3	1	*P. australis*
	2	*P. australis*, *A*. *sinensis* (Rare)
	3	*P. australis*, *S*. *salsa*, *S*. *viridis* (Very Rare)
	4	*P. australis*, *S*. *salsa*, *C. chinense* (Very Rare)
	5	*P. australis*, *S*. *salsa*
	6	*P. australis*, *C*. *chinense* (Rare), *S*. *viridis* (Very Rare)
	7	*P. australis*, *S*. *salsa*, *C*. *chinense* (Rare), *S*. *viridis* (Very Rare)
4	1	*P. australis*
	2	*P. australis*
	3	*P. australis*, *A*. *sinensis* (Rare)
	4	*P. australis*, *S. viridis* (Rare)
	5	*P. australis*, *S*. *salsa*, *C. chinense* (Very Rare)
	6	*P. australis*, *S*. *salsa*, *C. chinense* (Rare)
	7	*P. australis*, *S*. *salsa*, *C*. *chinense* (Rare), *S. viridis* (Rare)
5	1	*P. australis*
	2	*P. australis*
	3	*P. australis*, *S*. *salsa*, *S*. *viridis* (Very Rare)
	4	*P. australis, S*. *salsa*
	5	*P. australis*, *S*. *salsa*, *S*. *viridis* (Rare)
	6	*P. australis*, *S*. *salsa*, *S*. *viridis* (Rare), *C. chinense* (Very Rare)
	7	*P. australis, S*. *salsa*, *S*. *viridis* (Rare), *C. chinense* (Very Rare)

### Soil Total Petroleum Hydrocarbon Measurement

Three samples from the top 30 cm of soils were gathered from each quadrat. Soil total petroleum hydrocarbon (TPH) concentrations in the samples were measured using the infrared spectrophotometry method [39], [40]. In this method, dried (60°C/24 hour) soil samples (10 g) were extracted with 20 mL of carbon tetrachloride for 30 min by ultrasonication (40 kHz) at 40°C. The infrared absorption of the extract was measured at the band of 2930 cm^−1^. The amount of TPH was calculated as the difference between the amount of total TPH in the polluted soil minus the biogenic TPH content in the control [39].

### Multispectral Vegetation Indices (MVIs)

#### a. Normalized Difference Vegetation Index (NDVI)

This index has been proposed to eliminate seasonal sun angle differences and to minimize the effect of atmospheric attenuation [41].
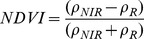
Where *NIR* and *R* denote the near-infrared band and red band, respectively.

#### b. Transformed Soil Adjusted Vegetation Index (TSAVI)

Many vegetation indices (such as TSAVI and SAVI2) rely on the existence of the soil line. TSAVI is a measure of the angle between the soil line and the line that joins the vegetation point and the intercept of the soil line [42].
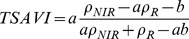
Where *a* and *b* represent the slope and intercept of the soil line, respectively. For *a* = 1 and *b* = 0, TSAVI is equivalent to NDVI. TSAVI equals 0 for bare soil and is close to 1 for high LAI.

The soil line is a linear relationship between bare soil reflectance observed in two different wavebands, and bare soil reflectance lies on a single line in the space generated by the wavelength bands. The soil line usually is derived from bare soil reflectance in red and near-infrared wavebands [42].The soil lines were obtained using broadband and narrowband data [42] of bare soils from 16 quadrats that we investigated in field.

The broadband soil line is:
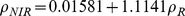



The narrowband soil line is:
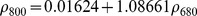
Where the numbers in the subscript represent the wavelengths used for calculation.

#### c. The second version of Soil Adjusted Vegetation Index (SAVI2)

SAVI2 uses the ratio of the intercept of the soil line *b* to its slope *a* as the soil adjustment factor [43].
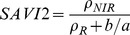



#### d. Atmospherically Resistant Vegetation Index (ARVI)

ARVI uses the difference in the radiance between the blue and the red bands to eliminate the atmospheric effect on the red band [20]. This correction enhances the resistance of ARVI to atmospheric effects (in comparison to NDVI).
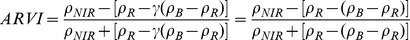
Where *γ* equals 1, this value is suitable for vegetated areas with small to moderate aerosol particle size and for arid regions with large particle size, and the subscript *B* denotes the blue band.

#### e. Optimization of Soil-Adjusted Vegetation Index (OSAVI)

OSAVI uses a different soil adjustment factor (*L* = 0.16) [44].
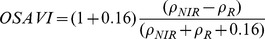



**Figure 3:**
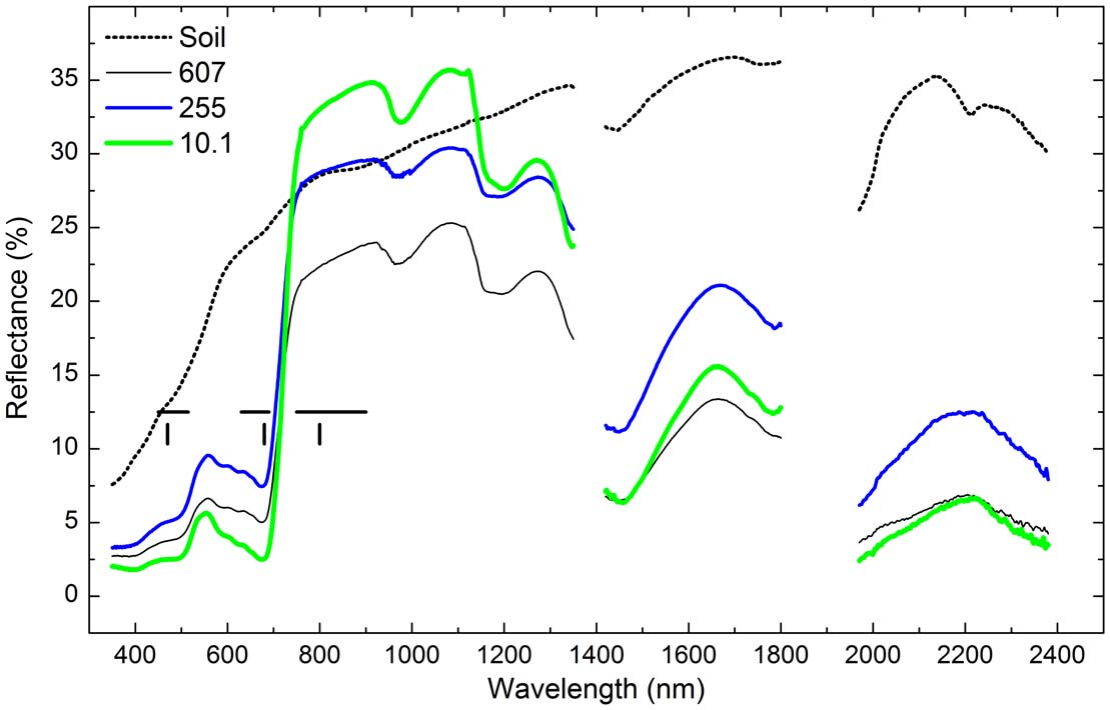
Reflectance of bare soil and reed communities with different soil TPH concentrations. Horizontal lines denoted broad wavebands used to calculate BMVIs. From left to right, they were blue (450–515 nm), red (630–690 nm) and near-infrared (750–900 nm). Vertical lines indicated wavelengths used to calculate NMVIs. From left to right, they were blue (470 nm), red (680 nm) and near-infrared (800 nm). The reflectance of bare soil was the average of 16 quadrats of bare soil. Each reflectance curve of vegetation was the average of 3 quadrats of reed communities at the same distance to the oil well in the same plot. The numbers in the legend were soil TPH concentrations (mg kg^−1^).

Multispectral vegetation indices were calculated with broadband and narrowband data in this study. Broadbands were blue (450–515 nm), red (630–690 nm) and near-infrared (750–900 nm), and narrowband were blue (470 nm), red (680 nm) and near-infrared (800 nm) (Figure 3).

### Hyperspectral Vegetation Indices (HVIs)

#### a. Pigment Specific Simple Ratio (PSSR) and Pigment Specific Normalized Difference (PSND)

The optimal individual wavelengths for the estimation of carotenoids are identified empirically as 470 nm [24].
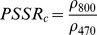


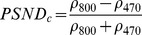



These indices provide a measure of the depth of the pigment absorption relative to the highly reflective near-infrared plateau. The near-infrared wavelength of 800 nm can be considered to minimize the effects of radiation interactions at the leaf surface and internal structures in mesophyll.

#### b. Modified Chlorophyll Absorption Ratio Index (MCARI)

MCARI is a modification of the Chlorophyll Absorption Ratio Index, which can minimize the effects of non-photosynthetic materials on the estimation of absorbed photosynthetically active radiation. The MCARI is the depth of the chlorophyll absorption at 670 nm relative to the reflectance at 550 nm and 700 nm [25].
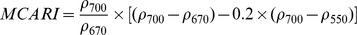



The ratio (*ρ*_700/_ *ρ*_670_) can minimize the combined effects of the underlying soil reflectance and the canopy non-photosynthetic materials [45].

#### c. Transformed Chlorophyll Absorption in Reflectance Index (TCARI)

The formula of TCARI is similar to that of MCARI with a different position of the ratio (*ρ*_700/_ *ρ*_670_) [45]. TCARI uses this ratio to counteract the background influence only on the difference (*ρ*_700 −_ *ρ*_550_).
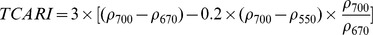



#### d. Maximum first derivative spectrum (deRES)

deRES was calculated as the maximum of the first derivative of the reflectance between 680 nm and 750 nm [26], [46].

#### e. Fifth-order polynomial fitting technique (fpnRES)

A fifth-order polynomial function is fitted to the reflectance spectrum between 680 and 750 nm [29].
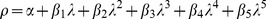
Where *λ* is the wavelength, *α*, *β* are parameters obtained by fitting. fpnRES was determined as the maximum of the first derivative of the fitted reflectance.

#### f. c2RES

Because there are two or more peaks in the first derivative reflectance of the red edge region, the peaks near 720 nm are identified as the main peak or dominant peak [21], [26]. c2RES was calculated as the maximum of the first derivative near 720 nm.

#### g. Sum of the first derivative (sumREA)

The sumREA was calculated as the sum of the first derivative of reflectances from 680 nm to 750 nm using data with a 1 nm interval from ASD software.

#### h. Gaussian function fitting technique (GauREA)

The first derivative of reflectance was fitted with the Gaussian function.
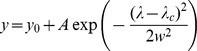
Where *y* is the first derivative of reflectance, and *y*_0_, *A*, *λ*_c_ and *w* are four parameters that determine the shape of the curve of the first derivative and are calculated by iteration.

Then the fitted curve was integrated as GauREA. This method is a modification of the inverted-Gaussian reflectance model that was used to fit the reflectance in the spectral region of 670–800 nm [28].

#### i. Sixth-order polynomial fitting technique (spnREA)

A sixth-order polynomial function is fitted to the reflectance spectrum between 680 and 750 nm [21].
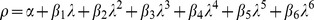
spnREA was calculated as the integral of the first derivative of the fitted reflectance.

The calculation of RES and REA were accomplished using OriginPro 8.5.1 (OriginLab Corporation, Northampton, MA, USA).

### Regression Analysis and Validation

Soil TPH concentrations, aboveground biomass, vegetation indices at the same distance from the oil wells in the same plot were averaged. The curve estimation module in IBM SPSS Statistics 19 was used to fit the relationship of aboveground biomass to soil TPH concentrations, and that of the reflectance at specific wavelengths, vegetation indices to soil TPH concentrations. Among 35 data of five wells, 30 data were used to fit above regression equations, other five data were used to validate the regression equations. The best equations were chosen from the linear, logarithmic, S, exponential, inverse and power models. The equations with a high R^2^, small root mean square error (RMSE) and good validation were regarded as good (Table 2).

**Table 2:** Regression equations of different spectral indices and soil TPH concentrations (y, mg kg^−1^) in the reed communities. *p*<0.001.

Category	Spectral indices (x)	Regression equations	*R* ^2^	Root mean squareerror (*RMSE*, mg kg^−1^)
BMVIs	Broadband TSAVI	y = 540.3−677.7x	0.70	109.3
	Broadband SAVI2	y = 527.0−283.9lnx	0.67	114.3
	Broadband ARVI	y = 461.5−591.0x	0.68	112.3
	Broadband OSAVI	y = 597.8−928.7x	0.68	112.7
NMVIs	Narrowband SAVI2	y = 519.9−271.3lnx	0.69	112.0
	Narrowband ARVI	y = 453.7−557.4x	0.70	109.4
	Narrowband OSAVI	y = 589.7−899.4x	0.69	110.6
HVIs	PSSRc	y = 776.2−327.1lnx	0.70	108.7
	PSNDc	y = 1017−1205x	0.71	107.5
	TCARI	y = 587.3−5136x	0.70	110.1
RES	fpnRES	y = −1207−242.2lnx	0.64	120.2
	c2RES	y = −1352−269.8lnx	0.67	112.9
REA	GauREA	y = −311.8−264.2lnx	0.64	119.1
	spnREA	y = −316.6−266.6lnx	0.65	118.7

To evaluate the regression equations performance, a measure used by Qiu *et al*. (1998) [47] was adopted to examine the correlation between the estimated and observed data. Employing this method, the observed and estimated data were used to derive a linear regression equation (*y* = a+b*x*), which was then compared with the 1:1 observed data line (*y* = *x*). Residual plots were used to evaluate the regression equations’ performance.

## Results

### Effect of Soil Oil Pollution on Aboveground Biomass of the Reed Community

**Figure 4:**
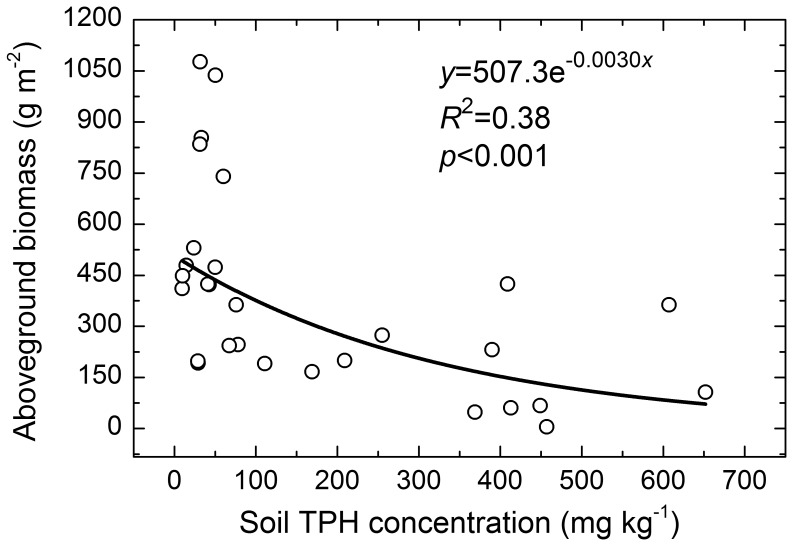
Relationship between soil TPH concentrations and aboveground biomass of the reed communities.

Soil TPH concentrations ranged from 9.45 mg kg^−1^ to 652 mg kg^−1^ with an average of 190 mg kg^−1^ and a standard deviation of 200 mg kg^−1^. Residual hydrocarbons were significantly and negatively correlated with the aboveground biomass of the reed community, which decreased from 1076.5 g m^−2^ to 5.3 g m^−2^ (Figure 4).

### Effect of Soil Oil Pollution on Reflectance of the Reed Community

Reflectance of reed community under three concentrations of soil TPH (Figure 3), which representing low, intermediate and high levels of oil pollution, shows that oil pollution decreased reflectance in the near-infrared between 800 nm and 1300 nm. However, reflectance in the other spectral bands did not follow the variations in TPH concentrations.

There were significant differences between reflectance of the reed community and that of bare soils (Figure 3). In the visible band and near-infrared band of 1400–2400 nm, the reflectance curve of the reed community was lower than that of bare soils. For the near-infrared band between 800 nm and 1300 nm, the difference in reflectance between the reed community and bare soils was smaller.

### Effect of Soil Oil Pollution on the First Derivative of Reflectance of the Reed Community

**Figure 5:**
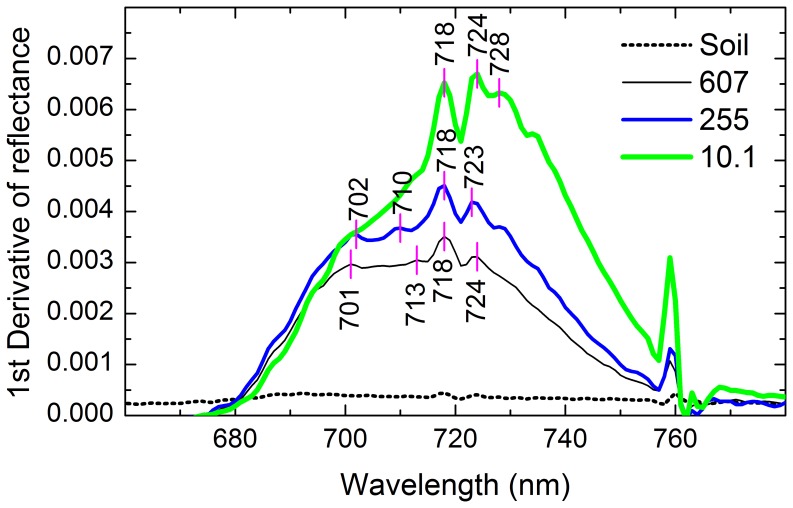
The first derivative of reflectance of bare soil and reed communities with different soil TPH concentrations. Numbers in the legend were soil TPH concentrations (mg kg^−1^). Vertical lines and numbers indicated the wavelengths of peaks (nm).

The first derivative of reflectance in the region of red edge distinguished bare soil and reed communities with different soil TPH concentrations (Figure 5). The first derivative of reflectance of bare soil was approximately a constant and close to zero, whereas that of the reed community was larger and had three or four asymmetric peaks. The first derivative of reflectance from three soil TPH concentrations shows that red edge slope and red edge area decreased with the increase in soil TPH concentrations.

### Performance of Spectral Indices for Estimating Soil Oil Pollution

**Figure 6:**
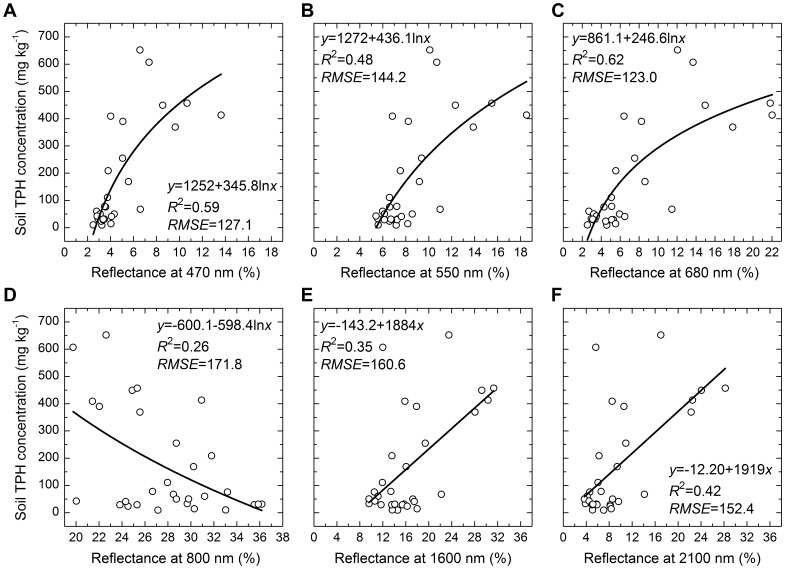
Performance of reflectance at specific wavelengths for estimating soil TPH concentrations. RMSE was the root mean square error. *p*<0.001.

Reflectances at specific wavelengths performed poorly for indicating soil oil pollution except for that at 680 nm, and reflectances at visible wavelengths performed better than that at near-infrared wavelengths (Figure 6).

**Figure 7:**
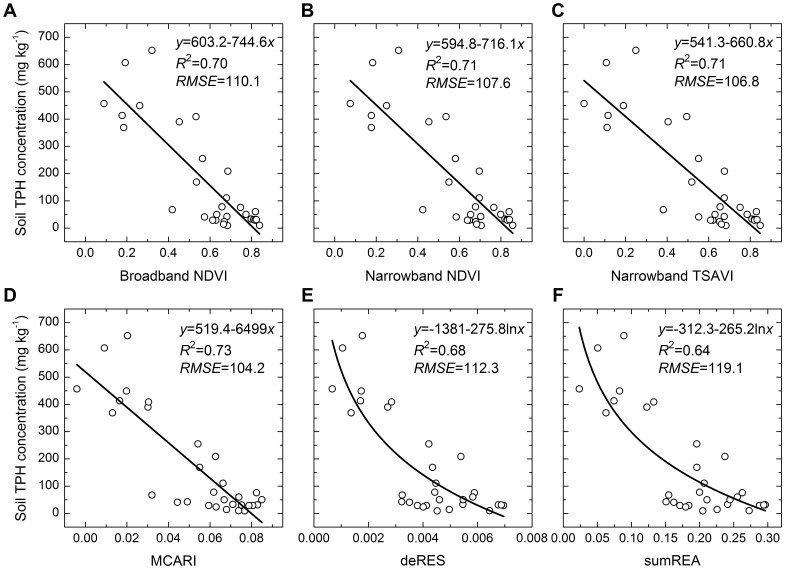
Performance of the leading vegetation indices for estimating soil TPH concentrations. NDVI, TSAVI, MCARI, deRES and sumREA denoted Normalized Difference Vegetation Index, Transformed Soil Adjusted Vegetation Index, Modified Chlorophyll Absorption Ratio Index, red edge slope calculated using maximum first derivative spectrum and red edge area calculated using the sum of the first derivative, respectively. RMSE was the root mean square error. *p*<0.001.

Compared with reflectances at single wavelengths, vegetation indices estimated soil TPH concentrations better, and most coefficients of determination of them exceeded 0.65 (Figure 7, Table 2). Compared with vegetations indices induced from red edge, other vegetation indices achieved comparable results and performed better. The results from red edge slope and red edge area were also comparable.

**Figure 8:**
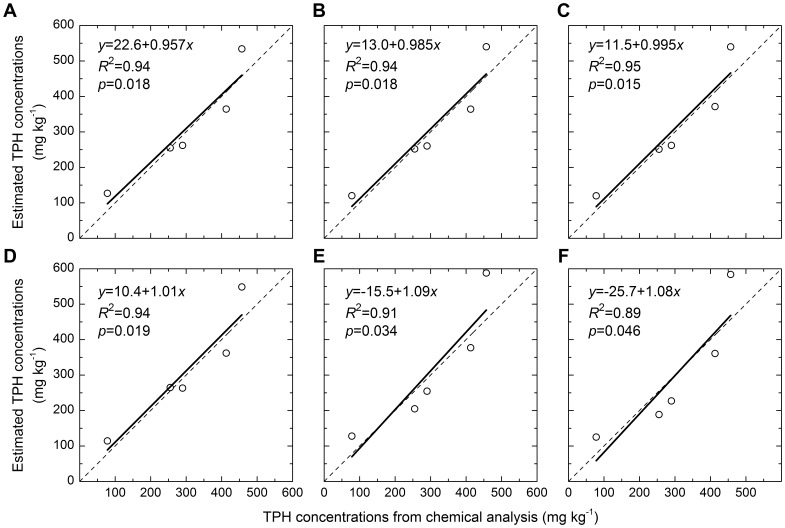
Comparison of observed and simulated TPH concentrations (mg kg^−1^). The dashed line showed the 1:1 relationship, the solid line, the fitted regression equations. A, B, C, D, E, F denoted the validation for the regression equations derived from broadband NDVI, narrowband NDVI, narrowband TSAVI, MCARI, deRES and sumREA, and abbreviations were the same as Figure 7.

**Figure 9:**
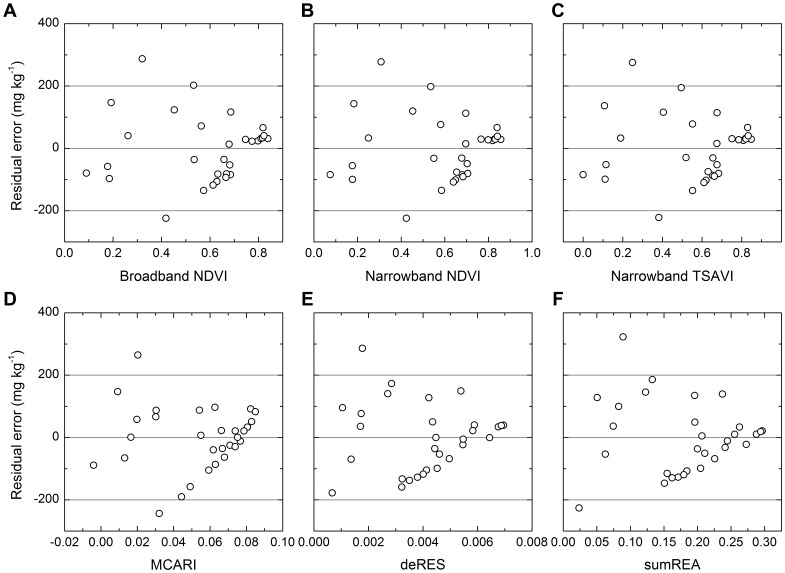
Residual plots for comparing prediction of TPH concentrations by different equations. Abbreviations were the same as **Figure 7**.

MCARI was the best among the 20 vegetation indices (Figure 7, 8, 9, Table 2). Among multispectral vegetation indices, the best estimations were given by NDVI and TSAVI, followed by narrowband ARVI. Narrowband NDVI and TSAVI were slightly better than their broadband versions. For hyperspectral vegetation indices, MCARI yielded the highest accuracy of prediction. PSNDc, PSSRc and TCARI could also estimate oil pollution well, and their coefficients of determination were larger than or equivalent to 0.70. Among red edge slope parameters calculated using different methods, deRES gave the best estimation of oil pollution. Red edge area values calculated using different methods achieved similar results (Figure 7, 8, 9, Table 2). It was clear from Figure 8 and 9 that the validation was in agreement with above results and residual error was acceptable.

## Discussion

### Persistence Times of Oil Pollutants in Soil

Hydrocarbons may persist in soils for 39 years and aboveground biomass was continuously decreased in hydrocarbon-contaminated areas [3]. Although it is recognized that reed dominated wetlands have potential for mitigating hydrocarbon pollution, reed biomass significantly decreased in the early stages of serious oil pollution [40], while very light short-term oil pollution led to an increase in reed biomass because of the added increment of carbon [48]. Further, ecological effects caused by long-term oil pollution were different from those resulting from short-term pollution [2]. In this study, there were residues of petroleum hydrocarbons around oil wells with a history of approximately 10 years, and aboveground biomass of the reed community decreased with increasing oil pollution (Figure 4). This result indicates that oil pollution resulted in significant change for the plant communities.

### Effects of Oil Pollution on Spectral Characteristics

Changes in the physiological status of plants caused by pollution and other environmental stresses can alter their spectral characteristics [33]. Also, reflectance at different wavelengths might show different sensitivities to these stresses [30]. Visible reflectance increased consistently in leaves subjected to eight stresses including pollution for six vascular plant species, while infrared reflectance was comparatively unresponsive to these stresses [30]. In this study, oil pollution altered the spectral characteristics of the reed community. Oil pollution increased the reflectance of light in the visible and near-infrared bands greater than that of 1400 nm, and decreased that in the near-infrared between 800 and 1300 nm (Figure 6). These effects were more significant for the reflectance of visible light than for that in the near-infrared (Figure 6). Thus, visible reflectance rather than that in the near-infrared was the more sensitive and reliable indicator of stresses.

Generally, changes of reflectance of plant canopy are mainly governed by leaf pigment concentrations, morphological and anatomical properties, water content and other biochemical properties [13], [14], and also can be modified by leaf area index, the amount of green biomass, canopy architecture and leaf angle distribution [15], [16]. Although most of these parameters were not measured in this study, aboveground biomass decrement (Figure 4) and reed communities coverage decrement (Figure 2) resulted from oil pollution might imply changes of leave properties such as leave pigment concentrations, water content and leaf angle distribution, and therefore leave reflectance were changed.

### Performance of Various Indices for Indicating Oil Pollution

Effects of soil on canopy reflectance caused a lower accuracy of prediction for estimating soil oil pollution using reflectance of single wavelengths compared with those using vegetation indices [22]. Our results also agree well with this conclusion (Figure 6, 7, Table 2). Therefore, it is necessary to mitigate the effect of soil on reflectance.

Most of the selected vegetation indices utilize red reflectance dominated by chlorophyll absorption and near-infrared reflectance governed by leaf scattering. These combinations effectively enhance the vegetation signal and suppress soil background effects [21]. Therefore, these indices more accurately characterize the spectral changes in canopy reflectance of vegetation under oil pollution. The MCARI and TCARI indices introduced a green band into their formulae and quantified the variations in the triangular area comprising the green peak, red reflectance minimum and near-infrared reflectance shoulder [21], [25], [49]. Therefore, these two indices also could estimate oil pollution well, and MCARI even produced the most accurate prediction of soil TPH concentrations in this study (Figure 7, 8, 9).

Multispectral vegetation indices and hyperspectral vegetation indices have also been used to estimate soil Zn concentrations. Compared to BMVIs, their narrowband variants showed a small improvement [17]. Similar results were found for the estimation of LAI and canopy chlorophyll density, with BMVIs yielding better predictions than their narrowband variants and hyperspectral vegetation indices [21]. In this study, all categories of vegetation indices achieved comparable accuracy of prediction for soil TPH concentrations (Figure 7, 8, 9, Table 2). These results indicate that NMVIs and hyperspectral vegetation indices had no absolute advantage over BMVIs.

Red edge parameters have the same theoretical foundation as the other vegetation indices consisting of red reflectance and near-infrared reflectance, because the red edge effect results from chlorophyll absorption of red light and leaf scattering of near-infrared light [21]. Because the first derivative of reflectance of bare soil was approximately a constant and close to zero, the derivative calculation of red edge parameters has the advantages of enhancing vegetation signals and suppressing the effects of bare soils [50]. Therefore, red edge slope and red edge area could estimate TPH concentrations with a higher accuracy than did the reflectance of single wavelengths (Figure 6, 7, 8, 9, Table 2) [17].

Compared to other vegetation indices, red edge parameters had a poorer performance in this study (Figure 7, 8, 9, Table 2). Firstly, this was dependent on their capacity to parameterize red edge. Polynomial and Gaussian function fitting have usually been used to calculate red edge slope and red edge area values. These methods often have the effect of smoothing out small spectral features that may contain important pollution-related information [32], [33]. In addition, because the first derivative curve in the region of red edge is asymmetric (Figure 5) and the Gaussian function is symmetric, Gaussian function fitting will average out such asymmetry [21]. Also, the existence of several peaks makes it more difficult to parameterize the red edge. For example, several plants from the greenhouse and the field also showed two peaks at around 700 and 725 nm [26]; A minor peak at 700 nm and a double peak around 720 nm were identified for annual bluegrass and perennial ryegrass [33]; One grass and two crops had four peaks at 702 nm, 718 nm, 725 nm and 735 nm in the red edge region [32]. Similarly, three or four peaks were present in the region of 700–730 nm in this study (Figure 5). However, methods of fitting assume that there is only a single peak in the red edge region. This might be another reason for the poor predictive capability of red edge in this study.

Therefore, traditional BMVIs such as NDVI still have a great potential for application in environmental monitoring because of their low cost, and more available data resources, such as Landsat TM, SPOT etc. compared with hyperspectral data [21], although Broadband NDVI has a lower *R* ^2^ (0.70) than that of MCARI (0.73). NMVIs and hyperspectral vegetation indices could be used to guide the design of field portable radiometer for vegetation monitoring [51], and only reflectance values at two or three wavelengths are required to quantify the growth status of vegetation and to estimate soil oil pollution.

### Conclusions

Residual petroleum hydrocarbon resulting from oil exploitation of approximately 10 years’ duration was associated with lowered aboveground biomass of plant communities dominated by reeds and altered their spectral characteristics. Spectral changes of the reed community under oil pollution provide an essential prerequisite for the estimation of soil oil pollution. Compared to reflectance at specific wavelengths, vegetation indices better characterized these spectral alternations. Therefore, they could be used to estimate soil TPH concentration with a higher accuracy. Among 20 vegetation indices, MCARI produced the highest accuracy of prediction and traditional broadband NDVI, one of the BMVIs, yielded a prediction similar to that of MCARI.

These results confirm that remote sensing has great potential for estimating oil pollution in soils over large areas under appropriate conditions. Traditional BMVIs still have great value for monitoring soil oil pollution when hyperspectral data are unavailable.
